# State Level Point-of-Sale Policy Priority as a Result of the FSPTCA

**DOI:** 10.3934/publichealth.2015.4.681

**Published:** 2015-10-16

**Authors:** Sarah Moreland-Russell, Todd Combs, Janice Jones, Amy A. Sorg

**Affiliations:** 1 Center for Public Health Systems Science, George Warren Brown School of Social Work, Washington University in St. Louis, St. Louis, MO

**Keywords:** Family Smoking, Prevention and Tobacco Control Act, Point of Sale Policy, state level tobacco control

## Abstract

The Family Smoking Prevention and Tobacco Control Act (FSPTCA) give the U.S. Food and Drug Administration (FDA) unprecedented power to regulate tobacco products. One of the most significant provisions of the law allows state and local governments to adopt and enforce tobacco control legislation restricting the time, place, and manner (but not the content) of tobacco advertising. However, there is still reluctance among states and localities for mass adoption of laws due to challenges associated with legal feasibility and lack of U.S.-based evidence in effectiveness. The Center for Public Health Systems Science conducted interviews with key tobacco control contacts in 48 states at two time points (2012 and 2014) since the passage of the FSPTCA to assess the influence of the law on point-of-sale policy development in their state tobacco programs. Logistic regression results show that point-of-sale policy importance is growing post-FSPTCA, and that key influencers of this importance are states' tobacco control histories and environments, including that related to excise taxes and smoke free air policies. The adoption of smokefree and tax policies has become commonplace across the U.S., and the quality and extent of these laws and prevailing political will increasingly impact the ability of states to work in emerging tobacco control policy areas including those directed at the point of sale.

## 1. Introduction

Tobacco use is one of the most catastrophic public health issues facing the world today. While current smoking among adults has declined from 20.9% in 2005 to 17.8% in 2013 an estimated 42.1 million adults in the U.S. currently smoke cigarettes.[1] Tobacco use is started and established primarily during adolescence,[2] making youth smoking rates particularly alarming. Each day in the U.S., more than 3,800 youth aged 18 years or younger smoke their first cigarette, and an additional 2,100 youth and young adults become daily cigarette smokers.[3],[4]

Tobacco control efforts aimed at reducing tobacco use and preventing initiation are combatted by the ever-present marketing and promotion of tobacco by the industry at the retail environment. The tobacco industry continues to spend most of its marketing budget in the retail environment.[5] Of the over $9 billion spent on advertising and promotions in 2012, price discounts paid to retailers and wholesalers—which ultimately reduce prices for consumers—comprised 85.1%.[6] The tobacco industry uses the strategic placement of products, price promotions and price discounts, signage and functional items containing product logos, and the products themselves to advertise and market tobacco products. The resultant advertising, promotion, and marketing of tobacco products in the retail environment increase youth and adult tobacco use and prompt impulse purchases.[5],[7]–[9]

In 2009, the Family Smoking Prevention and Tobacco Control Act (FSPTCA) provided the U. S. Food and Drug Administration (FDA) unprecedented power to regulate tobacco products. In addition, the FSPTCA granted state and local governments to explore, adopt, and enforce many retail interventions. Federal recognition of the need to adopt policies that address tobacco presence in the retail setting also allowed for POS policy strategies to be recognized as core strategies of tobacco control programming, along with: (1) raising cigarette excise taxes, (2) establishing smoke-free policies, (3) encouraging cessation, and (4) launching hard-hitting counter marketing campaigns.[5] Until the passage of the FSPTCA, interventions in the retail setting centered on restricting youth exposure and access to tobacco products. Now, thanks to the new federal authority granted to states and localities, communities across the U.S. are exploring additional retail interventions. These retail policy interventions fall into six policy areas and include those that (1) address licensing and restrict density, (2) use non-tax approaches to raise tobacco prices, (3) restrict product placement, (4) restrict advertising at the POS, (5) require health warnings, and (6)‘Other’ POS policies (e.g., Banning flavored other tobacco products and requiring minimum pack size). Table 1 provides examples of the types of policy strategies that fall into these six broad categories.

These retail policy interventions have set new precedents for reducing retailer presence. For example, San Francisco has capped the number of tobacco retail licenses issued at 45 in each of its 11 districts, and 80 municipalities in Massachusetts have banned tobacco sales in pharmacies.[10],[11] Traditional tobacco control strategies (e.g., implementing smoke free policies, increasing tobacco taxes, and enforcing laws prohibiting sales to minors), continue to be the core policy focus for states and communities seeking to reduce tobacco use. However, with the new provisions granted under FSPTCA and emerging evidence suggesting the effectiveness of retail interventions,[12],[13] the time has come for policy interventions in the retail environment to be considered alongside traditional interventions, particularly for communities that have made progress in other core areas of tobacco control.[14],[15]

Despite this recent progress, many questions and concerns among tobacco control partners remain. For instance, despite having authority to craft new point-of-sale policy, the legal feasibility of such interventions remains a barrier.[16] In addition, much of the evidence regarding effectiveness of retail policy interventions originates from international literature,[17]–[21] eliciting doubt regarding the applicability of such interventions in the U.S. Finally, competing priorities (e.g., resources directed at smokefree air or excise tax policy work) have delayed programs from exploring retail policy interventions.[22]

**Table 1.** Point-of-sale policy areas and example policies

Policy area	Example policies
Licensing & density	• Place a cap on the number of licenses in specific areas
	• Prohibit tobacco retailer operations around schools and parks
Nontax price increases	• Prohibit coupon distribution/redemption
	• Establish minimum price laws
Product placement	• Prohibit self-service product displays for all tobacco products
	• Ban product displays
Advertising & promotion	• Regulate in-store ad placement
	• Restrict outdoor advertising around schools and parks
Health warnings	• Require graphic health warnings at POS
	• Require posting of quitline information at POS
Other policies	• Raise minimum legal sales age
	• Require minimum pack sizes for cigars/cigarillos

Using a mixed method approach that includes logistic regression and thematic analysis, this paper investigates the perceived importance of retail interventions among state tobacco control programs. In addition, we explore the relationship between other state-level factors and perceived importance of retail interventions.

## 2. Materials and methods

Staff at the CPHSS conducted semi-structured interviews with state tobacco control staff at two time points: (1) April 2012 through September 2012 and (2) August 2014 through October 2014. Respondents were identified as primary state tobacco control contacts by the Centers for Disease Control and Prevention (CDC) Office for Smoking and Health. The survey was developed through extensive formative review of current literature and tobacco control expert input (e.g., state and local tobacco control staff, researchers, and legal experts). The survey was pilot tested in three states and modifications were made to the survey based on the pilot interviews and additional expert input. For each time point, 48 (96%) of state tobacco control program representatives agreed to complete the survey, and 46 (92%) of these participated in both (all states were surveyed at least once). For these 46 states, 29 were represented by the same individual each time, and one-third of the interviewees (n=17) for the second administration were different from the first administration. The study was approved by Washington University's Institutional Review Board.

Interviews were guided by a survey instrument developed to assess the level and types of POS policy activities occurring at the state and local levels. In addition to asking states which retail policy interventions they had implemented, we asked state tobacco control staff to rate the importance of focusing on retail interventions. Specifically, we asked respondents to assess whether POS policies had become more important to their state tobacco control program over the previous two years. In 2012 we asked, “Since the passage of the Family Smoking Prevention and Tobacco Control Act in 2009, would you say that point of sale policies are more or less important to your state tobacco control program?” In 2014, we asked, “Since we last spoke with your state in 2012, would you say that point of sale policies are more or less important to your state tobacco control program?” Answers were measured on a 5-point Likert scale ranging from -2, a lot less important; to 0, about the same; to +2, a lot more important. Many respondents provided additional comments explaining the rationale for their answer. These comments were coded using NVivo 10.[23] Two coders analyzed transcripts and emerging themes were identified to provide context for quantitative responses. The percent agreement between coders was above 90% for all applied codes, demonstrating excellent inter-rater reliability.

Quantitative analysis was conducted using the R statistical environment 3.1.1.[24] Logistic regression was used to explore the influence of traditional tobacco control program milestones on POS policy importance. Specifically, 2012 and 2014 data indicators for excise taxes, smokefree air policies, and tobacco control program funding were entered into the model. For excise taxes, we included three characteristics: the dollar amount, the years since the last excise tax increase, and the percentage of state tax revenue from tobacco. These data were acquired from the American Lung Association (ALA) and the U.S. Census Bureau.[25]–[27] The ALA smokefree score was used as an indicator for the state smokefree air policy, and funding was measured as the percentage of the CDC-recommended amount spent on tobacco control.[25],[26] To address the central question of whether POS policy work had become more important or not, we reclassified the POS policy importance responses as a binary variable, with zero indicating responses of “a little less important” or “about the same” and one indicating “a little” or “a lot” more important. We employed a pooled model after a fully-specified longitudinal logistic regression indicated no statistically significant state-fixed effects and a mixed-effects multilevel model with random effects for states showed results identical to the pooled model.

## 3. Results

**Figure 1.**
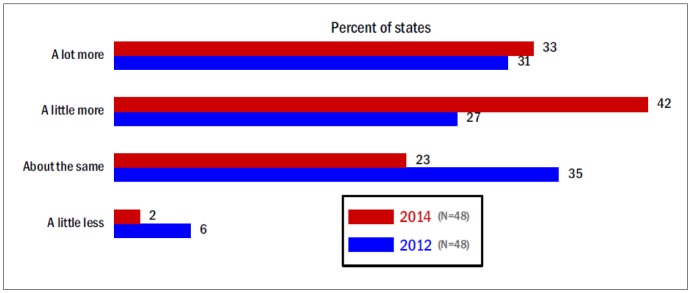
Change in importance of POS policy activity for state tobacco control programs from 2012 to 2014

All states were surveyed at least once, and of the 48 states surveyed in each 2012 and 2014, a majority reported that retail policy interventions had become a little or a lot more important to tobacco control efforts over the previous two years (Figure 1). Notably, data from the 2012 survey (three years after the passage of the FSPTCA) showed less than two-thirds (58%) claimed POS policies had become more important. Data from the 2014 survey (five years after the act) showed that three-quarters (75%) believed POS policy work as increasingly important tobacco control strategy.

Table 2 highlights qualitative analyses and provides some insight into participant scoring. Respondents who reported increased importance of retail policies cited a greater awareness around local authority and availability of tools to work on policy in this area: “We're doing more surveillance and using that evidence to educate” or “Now there's more information about [POS] policies and how to implement them.”[22] Among respondents who reported importance as being “about the same” or “a little less important”, several mentioned limited funding, competing priorities, and lack of capacity to do work in this area: “It's just not something that's high on our priority list because we have other things going on” or “Not that we don't know it's important but we have just not had the capacity to do something about [POS] issues the political climate right now is not such that we would get the support for some of these things.”[22] Political climate, preemption concerns, and fear of tobacco industry challenge were also mentioned as barriers.

**Figure 2.**
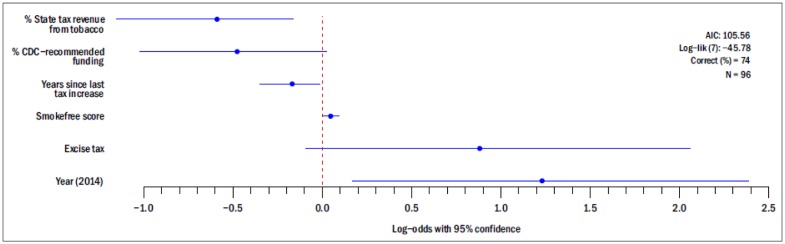
Smokefree policy success and excise tax increases influence POS policy importance

Figure 2 shows the results from a logistic regression for the binary response from both survey time points in the pooled model. Forty-six states are represented in both, while four only appear once (N = 96). Statistically significant predictors of POS policy importance are those with coefficients with confidence intervals that do not pass through zero (Figure 2) and include two of the three characteristics of excise taxes, percent of state revenue from tobacco tax and years since the last tax increase, along with the smokefree score and years since passage of FSPTCA. The percentage of state tax revenue from tobacco and the years since the last excise tax increase are negative indicating that as the proportion of state tax revenue from tobacco *or* the years since the excise tax was raised increases, the probability of POS policy being significantly more important decreases.

The ALA smokefree score and the dummy variable for 2014 are positive, indicating that as the smokefree score increases *or* that as more time passes since the FSPTCA, respondents are significantly more likely to say that POS policy had become more important. It is important to note the relatively large confidence interval for the estimate of the survey year, showing that while the effect of time passing since the passage of the FSPTCA is statistically significant, the magnitude of this effect is less certain. In addition, though not statistically significant, the percentage of CDC-recommended funding actually spent on tobacco control is almost globally negative, and the opposite is true for the amount of a state's excise tax, suggesting that both of these also may actually have influence on the importance of POS policies to tobacco control programs.

**Figure 3.**
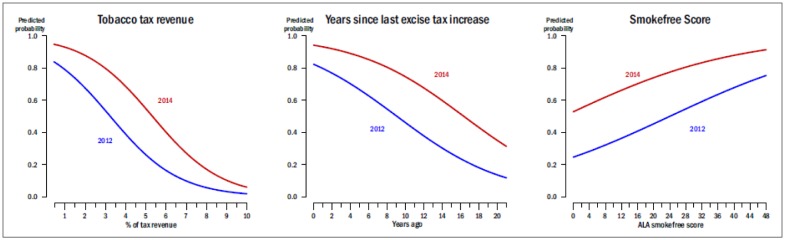
Effect of taxes and smokefree policy on POS policy importance

Figure 3 shows the changes in the predicted probability of POS policy importance across the ranges of the statistically significant effects holding all other variables at their means. All three plots show that the probability of POS policy importance was greater in 2014 than in 2012, suggesting that as more time passes since the FSPTCA, the POS policy work is becoming a more important tobacco control strategy. First, as tobacco taxes increase, POS policy work is less of a priority. States with relatively low tax revenue from tobacco in 2014 believed that POS policy strategies were gaining importance, while those with more substantial tax revenues were much less likely to report that POS policy work was increasingly important.

Next, states that have recently increased cigarette excise taxes are the most likely to see POS policies as increasingly important, and this likelihood steadily declines as years pass since excise tax increases. Finally, Figure 3 shows that smokefree scores are positively related to POS policy importance. As the ALA smokefree score increased, states became much more likely to report that POS policy work was a priority.

**Table 2.** Qualitative results regarding point of sale policy importance

Point of Sale Policy Importance	Main themes	Comments
A little less importantandabout the same	• Competing priorities for other tobacco control strategies • Fear of industry • Funding • Lack of political will • Lack of capacity	I think that we have so many other competing policy priorities, especially working on our smokefree policy and tax that the awareness is there, but it's still not a major focus, so I'd say it's probably the same. The political climate here is not such right now that it's something that we can even focus on. And then of course funding is always an issue so you have to try to make sure you prioritize and reach the things that you can. And we just haven't had the capacity to actually ... not that we don't know that it's important … we just have not had the capacity to be able to do that. It's something that I've got on my radar but we need more funding to make that a direction to go in.
A little or lot more important	• Achieved success in other tobacco control areas • Granted new authority to work in this area • Better access to good resources and tools • Greater evidence supporting POS policy interventions	Within the last year or two it's, like I said, one of the more prominent policies that were passed into … it's one that other states are working on as well. And the other two big ones, tobacco tax and Clean Indoor Air Act, we already have in place. Definitely more important because it's made very clear the role that public health can have and really making available local control opportunities for there to be good polices in local and state governments. So, yes, it's definitely much more on the forefront and surface of the level of importance. Yeah, there's more authority to work on this. And I just think the Center for Tobacco Products at the FDA actually has been I think providing an incredible amount of information, trying to get people to focus on this area. So I just think that there's increased awareness in tobacco control programs. But this is an area that we should be working on.

## 4. Discussion

This paper provides the first assessment of how states rank the importance of retail policy work since the passage of the FSPTCA. Our findings suggest that the FSPTCA has had a positive influence in the majority of states in providing the appropriate authority and impetus to work on retail policy. However, one quarter (25%) of states still report that the importance of retail policy as part of their state tobacco control efforts has not increased since the passage of the act mainly because of competing tobacco control policy priorities and lack of funding and capacity.

The findings from this paper highlight several potential factors affecting a state's ranking of POS policy as an important tobacco control strategy. POS policy work is more important for states that have already achieved success in the other core tobacco control strategies including increasing excise taxes and implementing smokefree policies. Possible explanations for this trend include that states with recent tobacco control policy success have more active tobacco control coalitions and/or policymaking bodies who are looking toward POS policies as a next step.[2] Also, in states where the excise tax has not been increased or that have not achieved smokefree policy success in the last 10–20 years, tobacco control may be a low political priority in general.

Taken together, these findings suggest that the POS is an important next step in tobacco control when traditional goals have been achieved or pursued to fullest extent given the political and economic environment. As the adoption of smokefree and tax policies becomes more commonplace across the U.S., the quality and extent of these laws and prevailing political will increasingly impact the ability of states to work in emerging tobacco control policy areas including those targeted at the POS. Conversely, as the evidence base grows and more POS policies are implemented and evaluated, new avenues of research can investigate how successes in this newer policy area might be leveraged to reenergize and strengthen traditional efforts like smokefree air laws.

Some limitations of this study should be acknowledged. First, the POS policy importance ranking was reported by one state-level tobacco control staff member and may not completely reflect the belief of tobacco control stakeholders within the state. Although respondents were typically managers with substantial knowledge of tobacco control activity within their state, some differences in respondent awareness and experience level is to be expected. In addition, in 17 of the states participating in both the 2012 and 2014 survey, different staff members interviewed from administration to administration. Next, different measurement strategies for modeling increased POS policy importance might produce different results. The findings that percentage of CDC-recommended funding and excise tax amount were not statistically significant predictors is puzzling, since most relatively well-funded and high-tax tobacco control environments anecdotally also have relatively high POS policy activity. Cost-of-living adjusted excise tax levels and program funding per smoker or youth tobacco user might produce a more accurate picture of the relationships between POS policy importance and funding and tax.

The policies, partnerships, and intervention activities that occur at the state and community levels drive social norm and behavior change within states and across the nation.[5] State programs can set the political agenda and keep tobacco issues before the public, promoting community and policy maker buy-in and support, and ultimately informing policy change. Though the FSPTCA provided an unprecedented opportunity for state-and local-tobacco control to enhance and continue efforts within the retail setting, the opportunity was delivered without active guidance or immediate funding to support movement in implementation of these policies. Because of the immense presence of industry in stores where people representing all ages, race and ethnicities frequent, there is a great need from federal agencies and from academia to support states in their efforts to prioritize this work.
